# Prognostic effect of osteoprotegerin in patients with ischemic stroke: A systematic review and meta-analysis

**DOI:** 10.1371/journal.pone.0303832

**Published:** 2024-05-31

**Authors:** Linlin Pang, Hongyu Lin, Xinxian Wei, Wenxin Wei, Yu Lan

**Affiliations:** 1 Department of Neurology, Minzu Hospital of Guangxi Zhuang Autonomous Region, Nanning, Guangxi Zhuang Autonomous Region, China; 2 Department of Neurology, Red Cross Hospital of Yulin City, Yulin, Guangxi Zhuang Autonomous Region, China; Istanbul Health and Technology University, Faculty of Medicine, TURKEY

## Abstract

**Background:**

Osteoprotegerin (OPG) is supposed to participate in the development of atherosclerosis and cardio-cerebrovascular disease. However, the results of research on relationship between OPG and ischemic stroke (IS) are controversial. Therefore, we carried out the first systematic review and meta-analysis to evaluate prognostic effect of osteoprotegerin in patients with IS.

**Methods:**

We comprehensively searched databases of PubMed, Embase, and the Cochrane Library through 21 August 2023 to identify observational studies that evaluated effect of OPG on poor functional outcome (modified Rankin Scale [mRS] Score of 3–6) and mortality in patients with IS. Adjusted odds ratios (aOR) with a 95% confidence interval (CI) of each included study were used as much as possible to assess the pooled effect.

**Results:**

Five studies that enrolled 4,506 patients in total fulfilled our inclusion criteria. Three studies were included in the pooled analysis for each endpoint since one of the included studies had provided data on poor functional outcome as well as mortality. OPG was neither associated with poor functional outcome (aOR 1.29, 95% CI 0.90–1.85) nor with mortality (aOR 1.57, 95% CI 0.90–2.74) in patients with IS.

**Conclusions:**

There is insufficient evidence to demonstrate the correlation between OPG and mortality or poor functional outcome in IS patients. OPG cannot be applied to predict worse neurological function in IS patients based on the current evidence.

## Introduction

Ischemic stroke (IS), a major cause of disability and mortality worldwide as well-known, has 7.6 million new cases annually and can result in approximately 3.3 million deaths each year [1].IS is classified as an inflammatory disease, with inflammation playing a crucial role in all stages of its pathophysiology, including development, acute damage cascades, and chronic course progression [2]. Prior research has indicated that certain inflammation biomarkers are associated with a heightened risk of IS [2–4], and osteoprotegerin (OPG) may serve as a potential predictor for the development of IS [3, 4]. OPG, a soluble member of the tumor necrosis factor (TNF) receptor superfamily that acts as a decoy receptor for the receptor activator of nuclear factor-κB ligand (RANKL) and TNF-related apoptosis-inducing ligand (TRAIL), has been primarily investigated in the realm of bone turnover and metabolism [5, 6]. In recent decades, OPG has been proposed as a modulator of inflammation including the regulation of lymphocytes and apoptosis [7] and as a participant in the pathogenesis of atherosclerosis in cardiovascular disease [8].

Emerging evidence suggests a correlation between circulating OPG levels and an elevated risk of coronary artery disease, heart failure and stroke [9]. Additionally, OPG levels are proposed to function as a potential prognostic biomarker for unstable angina, myocardial infarction and heart failure [9]. Nevertheless, the impact t of OPG on patients with ischemic stroke remains constrained and inconclusive. While certain studies have demonstrated an association between OPG and the occurrence of IS [3, 10, 11], others have not [7, 12]. Moreover, a significant association has been observed between elevated OPG levels and poor functional outcome [9, 13, 14] as well as increased mortality [9, 15, 16]. However there is also evidence indicating that serum levels of OPG and RANKL do not exhibit a significant correlation with radiological or clinical parameters and scores in patients with ischemic stroke [17]. Therefore, we conducted the initial systematic review and meta-analysis of existing evidence to evaluate the prognostic effect of OPG in patients with IS.

## Methods

The current systematic review and meta-analysis were conducted in accordance with the guidelines outlined in the Preferred Reporting Items for Systematic Review and Meta-Analysis (PRISMA) statement [18] and had not been registered protocol. Patient consent or ethical approval is not required as the data and analyses utilized in this study were derived from previously published studies.

### Literature search

We performed a systematic and comprehensive electronic search in PubMed, Embase, and the Cochrane Library from inception through 21 August 2023, without any geographical or linguistic restrictions. The search terms utilized in the literature search were listed as following: (‘osteoprotegerin’ or ‘osteoclastogenesis inhibitory factor’ or ‘tumour necrosis factor receptor 11b’ or ‘follicular dendritic cell derived receptor 1’ or ‘FDCR 1 Protein’), and (‘ischemic stroke’ or ‘ischaemic stroke’ or ‘cerebral infarction’ or ‘brain infarction’). MeSH or Emtree terms and their variations were combined for the search. Detailed searching procedures are shown in S1 Table (Embase), S2 Table (PubMed) and S3 Table (the Cochrane Library). Additionally, a manual examination of the bibliographies of pertinent and incorporated studies was conducted to identify any additional potentially eligible papers.

### Selection criteria

Two authors (L.-L. P. and H.-Y. L.) independently undertook the initial search, deleted duplicate records, and subsequently excluded irrelevant literature after reviewing titles and abstracts. The remaining potentially relevant literature was then assessed in full-text format to determine eligibility. We resolved discrepancies or disputes by discussion and consensus.

Studies meeting the following criteria were considered for inclusion: (1) case—control studies, cohort studies, or retrospective studies; (2) studies examining the relationship between circulating OPG levels and poor functional outcome (modified Rankin Scale [mRS] Score of 3–6) or mortality in ischemic stroke; and (3) studies comparing the highest quartile OPG level with the lowest quartile OPG level and providing either the odds ratio (OR) or adjusted odds ratio (aOR) directly, or containing data that allowed for the calculation of OR or aOR. Exclusion criteria included studies that exclusively recruited cases, published abstracts, animal experiments, reviews, comments or letters. The study with the largest sample size or the most comprehensive data was chosen among studies containing duplicate data.

### Data extraction

One author (L.-L. P.) extracted essential data from articles included in the study using a standardized data-extraction form, while two other authors (X.-X. W. and W.-X. W.) independently verified the accuracy of the extracted data. The collected data encompassed various elements, such as the primary author, publication year, country of origin, study design, duration of research, inclusion and exclusion criteria, sample size, and outcome data. Data validation and discrepancies were addressed through collaborative discussions with coauthors.

### Quality assessment

The quality of the included studies was evaluated utilizing the Newcastle-Ottawa Scale (NOS) [19], a widely recognized and reliable tool for assessing the quality of observational studies. This scale evaluate study quality from the aspect of eight items across three key areas: selection of the participants, comparability of the participants and outcomes. Study quality is categorized as low, moderate, or high based on scores falling within the ranges of 1–3, 4–6, and 7–9, respectively.

### Statistical analysis

When both OR and aOR, along with their corresponding 95% confidence intervals are reported simultaneously in a single study, the preference is to utilize aOR for evaluating the pooled effect in meta-analysis due to its adjustment for known potential confounding factors. This adjustment enhances the accuracy and credibility of the pooled results. Heterogeneity among studies was assessed using the *I*^*2*^ statistic, with a value greater than 50% indicating significant heterogeneity [20]. Based on the calculated *I*^*2*^ value, a fixed effects model was selected when *I*^*2*^ was less than 50%, while a random effects model was chosen when *I*^*2*^ exceeded 50%, taking into account the variations among the included studies. Sensitivity analysis was performed by systematically excluding each study to evaluate its impact on the pooled outcome. Subgroup analysis, publication bias assessment, and meta-regression analysis were not performed in the present meta-analysis due to the limited number of studies included in the pooled effect estimation for each endpoint. A statistically significant result was determined by a p-value less than 0.05.. All statistical analyses were carried out using Stata software version 12.0 (Stata Corp LP, College Station, TX, USA).

## Results

### Study selection and characteristics

**Fig 1:**
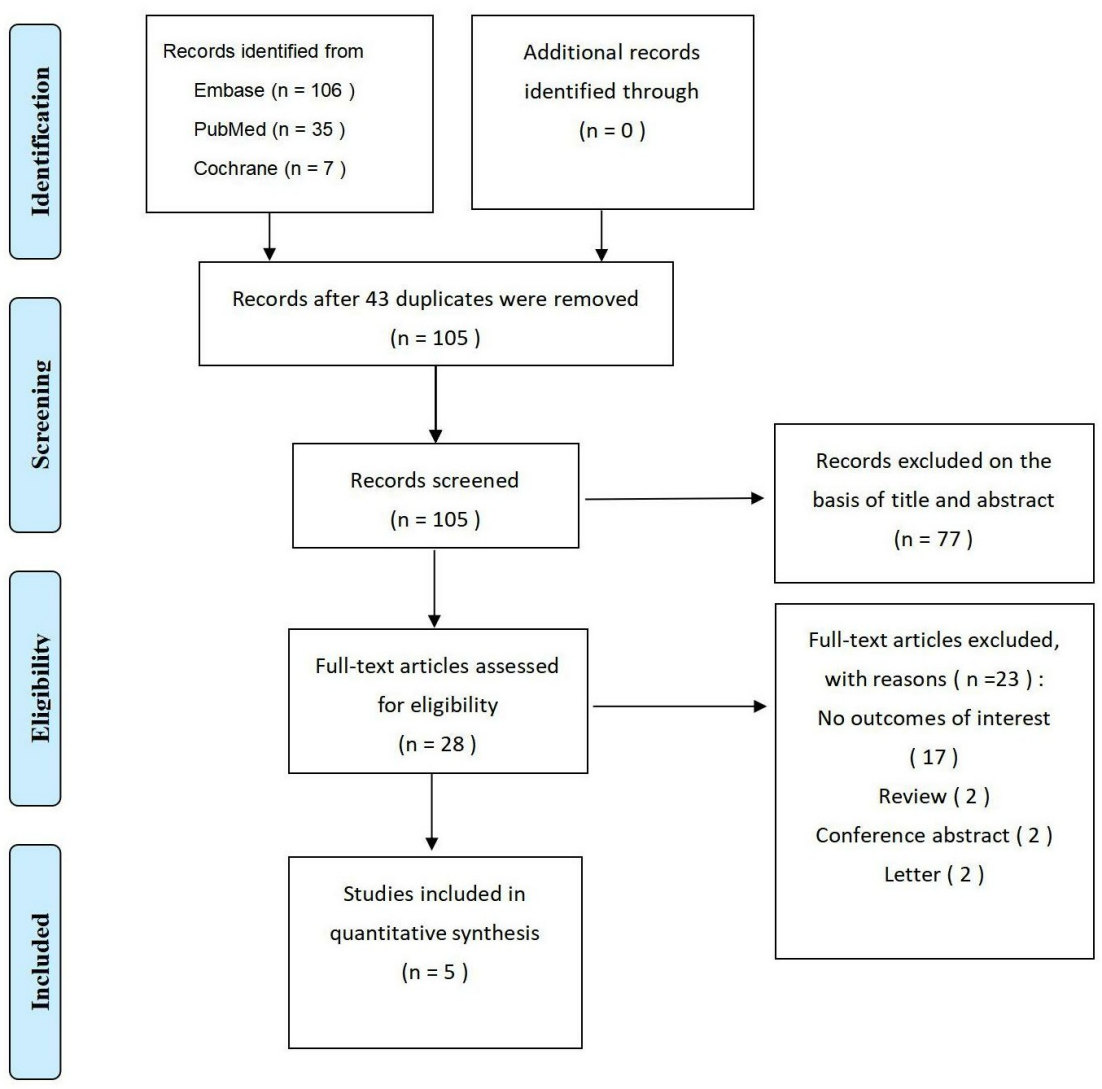
Flowchart of screening for literature selection.

The PRISMA statement flowchart illustrates the steps of identification, screening, and selection of eligible studies (Fig 1). We identified 148 citations from the initial electronic database search. Following removing duplicates and screening the titles and abstracts, 28 articles were deemed potentially suitable for inclusion. After conducting a comprehensive full-text review, five studies [9, 13–16] were ultimately included in the meta-analysis. The quality assessment of the included studies was performed using NOS (see Table 1; detailed scores provided in S4 Table). One study received a score of 8 stars [13], while the remaining four studies received scores of 7 stars [9, 14–16], indicating a high level of quality for each study.

**Table 1:** Characteristic of included studies that evaluated OPG levels and poor functional outcome and mortality in the meta-analysis.

Study	Country	Design	Research time	Inclusion criteria	Main exclusion criteria	Sample size	Poor functional outcome	Mortality	Adjustment for Covariates	NOS scroe
Song et al 2012	Korea	prospective cohort study	NA	AIS within 7 days after symptom onset	a history of any malignancy, a bone fracture within 2 months, an autoimmune disease, or rare cause of stroke	172	1.004 (1.001–1.007)^a^	-	age, sex, hypertension, previous stroke, and infarction volume	8
Zhu et al 2022	China	prospective cohort study	August 2009 to May 2013	AIS within 48 hours after symptom onset	systolic BP ≥220 or diastolic BP ≥120 mm Hg, severe cerebrovascular stenosis (≥70%), acute myocardial infarction or unstable angina, atrial fibrillation, severe heart failure, resistant hypertension, aortic dissection, deep coma, or receiving intravenous thrombolytic therapy	3,490	1.40 (1.05–1.88)^a^	2.05 (1.04–4.08)^a^	age, sex, time from onset to hospitalization, current smoking, alcohol consumption, systolic BP, white blood cell counts, and NIHSS score at baseline, dyslipidemia, diabetes, history of hypertension, history of coronary heart disease, family history of stroke, use of lipid-lowering drugs before stroke onset, use of antihypertensive medications before stroke onset, ischemic stroke subtypes, and receiving immediate BP reduction	7
Wajda et al 2019	Poland	prospective cohort study	January 2013 to August 2015	first episode ischemic stroke	hemorrhagic stroke, a history of cancer, apparent inflammation, and impairment in activities of daily living before the stroke	240	-	1.084 (1.036–1.134)^a^	NA	7
Jensen et al 2010	Denmark	prospective cohort study	August 2003 to October 2004	AIS	overt ischaemic heart disease, current atrial fibrillation, onset of stroke symptoms >7 days before admission, technical reasons or lack of compliance	244	-	2.3 (1.1–4.9)^a^	age, Scandinavian Stroke Scale score, C-reactive protein, troponin T > 0.03 ug L^-1^, heart and/or renal failure	7
Park et al 2022	Korea	retrospective observational study	April 2014 to December 2020	AIS patients who underwent endovascular thrombectomy	uncommon stroke risk including cancer or an autoimmune disease, bone fracture within the last two months	360	2.121 (1.089–4.191)^a^	-	sex, body mass index, age, NIHSS, DM, thrombolysis methods, number of trials for thrombectomy, successful recanalization, any hemorrhagic transformation, blood glucose level at admission, hemoglobin, total cholesterol, WBC, C-reactive protein, and vitamin D 25	7

AIS indicates acute ischemic stroke; BP, blood pressure; NIHSS, National Institute of Health Stroke Scale; DM, diabetes mellitus; WBC, white blood cell; NA, not available.

^a^ Adjusted odd ratio (95% confidence interval).

### Study characteristics

The primary attributes of the included studies are outlined in Table 1, encompassing research conducted in China, Korea, Poland and Denmark. These studies were published between 2009 and 2023, with sample sizes ranging from 240 to 3,490, totaling 4,506 patients. The majority of enrolled patients exhibited symptoms of acute ischemic stroke within 7 days, including those who underwent endovascular thrombectomy in the investigation conducted by Park *et al* [14]. One study provided aOR and 95% confidence intervals for both poor functional outcome and mortality [9], while two studies reported only poor functional outcome [13, 14], and two other studies reported only mortality [15, 16].

### Outcomes

**Fig 2:**
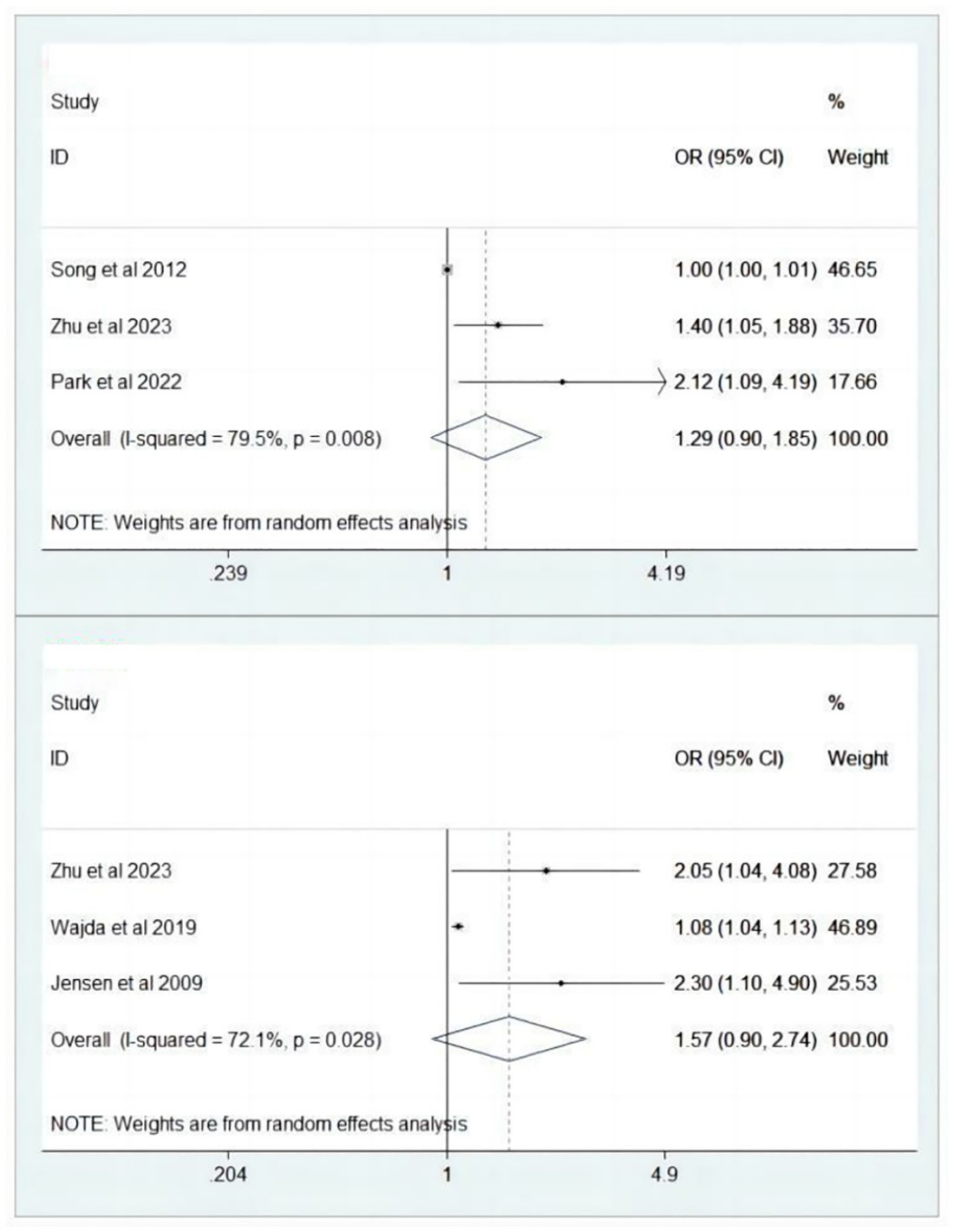
Forest plot illustrating the association between osteoprotegerin (OPG) and poor functional outcome (Fig 2A) and mortality (Fig 2B) in ischemic stroke patients.

**Fig 3:**
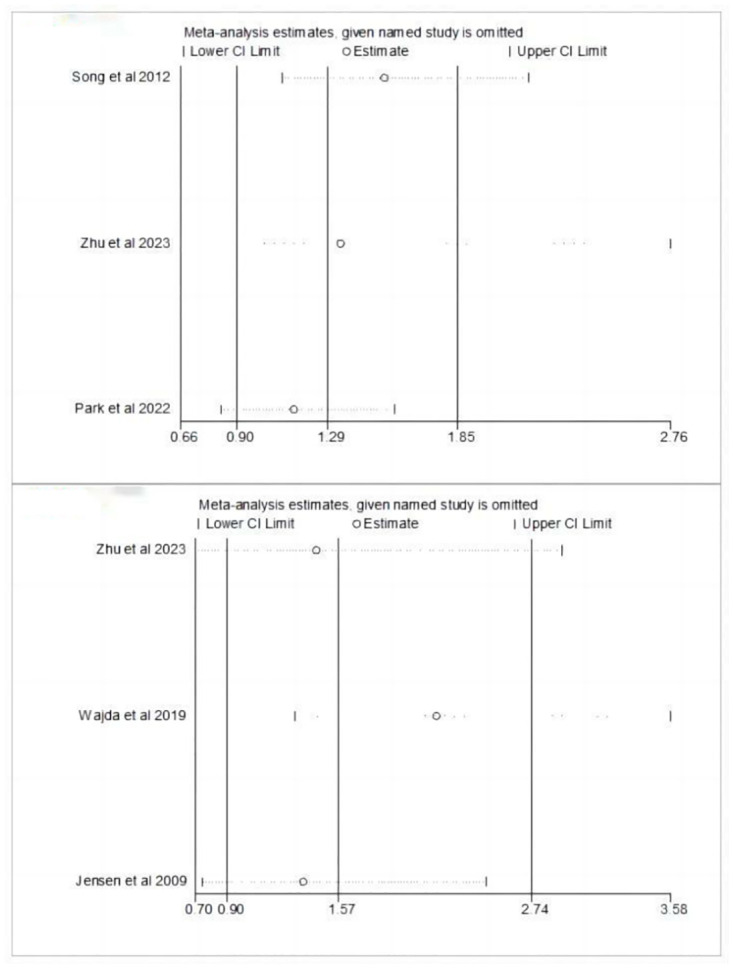
Sensitivity analysis for the robust assessment of poor functional outcome (Fig 3A) and mortality (Fig 3B).

While individual studies indicated a positive correlation between OPG and poor functional outcome [9, 13, 14], the pooled analysis revealed no significant association between OPG and poor functional outcome in patients with ischemia stroke (aOR 1.29, 95% CI 0.90–1.85). The high *I*^*2*^ statistic of 79.5% indicated a considerable level of heterogeneity among the studies (Fig 2A). During sensitivity analysis, each individual study was sequentially excluded, leading to the identification of Song et al’s study [13] as a significant contributor to heterogeneity and a chang in the outcome regarding concomitant use (aOR 1.53, 95% CI 1.09–2.15) (Fig 3A).

Similarly, a lack of significant correlation was observed between OPG levels and mortality in patients with ischemic stroke (aOR 1.57, 95% CI 0.90–2.74) (Fig 2B), despite the individual findings of three included studies indicating a positive association between OPG levels and mortality [9, 15, 16]. Sensitivity analysis revealed that the study by Wajda et al. [13] contributed to heterogeneity and resulted in a reversal of the outcome when considering concomitant use (aOR 2.16, 95% CI 1.30–3.58) (Fig 3B).

## Discussion

### Main outcome

After reviewing the existing literature, our analysis indicates that there is no significant association between circulating OPG levels and poor functional outcome or mortality in patients with ischemic stroke. Further examination of this finding is warranted, as it may not be consistently supported in sensitivity analyses.

### Relationship and potential mechanisms between OPG and IS

Osteoporosis and cardiovascular disease, despite appearing as distinct conditions, are intricately linked due to shared risk factors and a common feature of low grade chronic inflammation [8]. OPG, originally identified by Simonet et al. as a pivotal modulator in bone metabolism [21], has garnered increasing attention for its involvement in vascular disease and calcification in recent years. Nevertheless, the precise mechanisms by which OPG operates within the vascular system remain elusive, and the findings of pertinent investigations are subject to debate. Numerous clinical researches have demonstrated a positive correlation between elevated levels of circulating OPG and the incidence of various cardiovascular diseases, such as coronary artery disease, heart failure, unstable angina, peripheral artery disease, symptomatic carotid stenosis, and vulnerable carotid plaques [22]. OPG was found to be independently correlated with traditional risk factors of atherosclerosis, subclinical peripheral atherosclerosis, and clinical atherosclerotic disease including ischemic heart disease and ischemic stroke [23]. Findings from the 4^th^ Copenhagen City Heart Study [24] and the Tromsø Study [25] indicated that elevated levels of circulating OPG were predictive of increased risk for ischemic heart disease, ischemic stroke, and all-cause mortality in general population. Similarly, studies have demonstrated elevated levels of circulating OPG in IS patients compared to the general population [11, 26, 27]. Moreover, elevated levels of OPG have been found to be associated with incident [10, 11, 28], severity at admission [10, 27, 28], poor functional outcome [9, 13, 14] and mortality [9, 15, 16] following an IS event. This association is supported by evidence linking elevated plasma OPG levels to vulnerable carotid plaques [29], larger infarctions [13], higher National Institutes of Health Stroke Scale scores at admission [13] and the occurrence and severity of hemorrhagic transformation [30]. The potential mechanisms by which OPG may be linked to the development of inflammatory diseases such as atherosclerosis are multifaceted. Firstly, OPG has been shown to upregulate the expression of intercellular molecule-1, vascular adhesion molecule-1, and E-selectin on endothelial cells, leading to increased leukocyte adhesion, a critical early event in endothelial dysfunction [31]. This suggests that OPG palys a important pro-atherosclerotic role in vascular diseases. Secondly, OPG has been found to significantly enhance the RANKL-stimulated effect on matrix metalloproteinase (MMP) activity, particularly at specific OPG/RANKL molar ratios [32]. Additionally, high concentrations of OPG have been shown to directly induce MMP activity directly [32]. MMP is widely recognized for its role in promoting extracellular matrix degradation, potentially contributing plaque instability destabilization and increasing the incidence of vascular diseases. Thirdly, OPG has been shown to facilitate the anchoring of ultra large von Willebrand factor (vWF) multimers at the site of vascular injury, thereby promoting localized thrombus formation [33].

In contrast to the aforementioned perspective, there are studies that offer a divergent viewpoint, as demonstrated by the results of our research article. To date, only three studies have presented divergent perspectives on the clinical implications for IS patients. One such study, which focused on a cohort of 490 white women aged 65 years and older, found no significant association between OPG levels and baseline bone mineral density or with subsequent strokes or fractures [7]. Additionally, Nybo and colleagues conducted a nested case-control study within a large population of 57,053 individuals in Denmark, concluding that plasma OPG concentrations were not associated with an increased risk of ischemic stroke [12]. Another separate investigation examing the impact of serum osteopontin, OPG and RANKL in individuals with acute ischemic stroke indicated that OPG did not demonstrate any significant association with radiological or clinical parameters and scores [17], although detailed data was regrettably unavailable. There are two potential explanations for the adverse outcomes observed in these study. Firstly, elevated serum OPG levels in humans may represent a reactive response rather than a causal factor in the development of atherosclerosis or vascular calcification [7]. Secondly, cardiovascular disease may potentially benefit from OPG due to its inhibitory effects on vascular calcifications at higher concentrations [12]. Research involving animals has also shown that OPG exerts a protective influence against vascular calcification by potentially inhibiting alkaline phosphatase activity [34–37]. Hence, the absence of correlation between OPG and IS probably repesents the underlying circumstances.

### Strengths and limitations

There are some limitations in our study. First of all, the final result lacks robustness in sensitivity analysis, potentially due to the inclusion of rare included literature, variations in study populations, sample sizes, study designs and adjusted confounding factors. Secondly, we had not performed subgroup analysis, publication bias and meta-regression analysis due to the scarcity of included literature, preventing further identification of factors influencing result robustness. Furthermore, the establishment of a causality relationship between OPG and IS is hindered by the reliance on observational studies in the existing literature, as well as the lack of clarity regarding the exact pathophysiological mechanisms of OPG. Therefore, additional well-designed researches are necessary to investigate and address these unresolved issues.

Conversely, our study possesses several strengths. To the best of our understanding, the present meta-analysis is the inaugural publication to thoroughly assess the relationship between OPG and IS. We employed adjusted ORs from each incorporated literature to aggregate the findings in order to mitigate the influence of confounding factors, thus enhancing the accuracy and credibility of our results.

## Conclusions

In conclusion, the exising evidence does not conclusively establish a correlation between OPG levels and mortality or poor functional outcome in patients with ischemic stroke. Therefore, OPG cannot currently be utilized as a predictive marker for adverse neurological outcomes in this patient population. Additional research is necessary to elucidate the precise pathophysiological mechanisms and prognostic implications of OPG in ischemic stroke.

## Supporting information

S1 Checklist PRISMA 2020 checklist. (DOCX)

S1 Table Searching procedure on Embase. (DOCX)

S2 Table Searching procedure on PubMed. (DOCX)

S3 Table Searching procedure on the cochrane library. (DOCX)

S4 Table Quality assessment of included studies by newcastle-Ottawa scales. (DOCX)

## Abbreviations

AIS: acute ischemia stroke
aOR: adjusted odds ratios
BP: blood pressure
FDCR: follicular dendritic cell derived receptor
IS: ischemia stroke
MMP: matrix metalloproteinase
mRS: modified Rankin Scale
NOS: Newcastle-Ottawa Scale
NR: not reported
OPG: osteoprotegerin
OR: odds ratios
PRISMA: Preferred Reporting Items for Systematic Review and Meta-Analysis
RANKL: nucleus factor-κB ligand
TNF: tumor necrosis factor
TRAIL: TNF-related apoptosis-inducing ligand
vWF: von Willebrand factor
