# Supplementary material for: Prognostic effect of osteoprotegerin in patients with ischemic stroke: A systematic review and meta-analysis
Source: PLoS One. 2024 May 31;19(5):e0303832. doi: 10.1371/journal.pone.0303832 (PMC11142426; doi:10.1371/journal.pone.0303832)
Supplement: S1 Table — (DOCX) [file pone.0303832.s002.docx]

S1 Table. Searching procedure on Embase

| Step | Query | Results |
| --- | --- | --- |
| 1 | ‘ischemic stroke’/exp | 28,443 |
| 2 | ischemic stroke:ab,ti | 169,658 |
| 3 | ischemic strokes:ab,ti | 20,739 |
| 4 | ischaemic stroke:ab,ti | 22,773 |
| 5 | ischaemic strokes:ab,ti | 3,435 |
| 6 | cerebral infarction:ab,ti | 46,037 |
| 7 | cerebral infarctions:ab,ti | 5,630 |
| 8 | ‘brain infarction’/exp | 90,586 |
| 9 | brain infarction:ab,ti | 66,922 |
| 10 | brain infarctions:ab,ti | 7,312 |
| 11 | #1 OR #2 OR #3 OR #4 OR #5 OR #6 OR #7 OR #8 OR #9 OR #10 | 289,879 |
| 12 | ‘osteoprotegerin’/exp | 12,944 |
| 13 | osteoprotegerin:ab,ti | 9,110 |
| 14 | osteoclastogenesis inhibitory factor:ab,ti | 1,012 |
| 15 | tumour necrosis factor receptor 11b:ab,ti | 11 |
| 16 | follicular dendritic cell derived receptor 1:ab,ti | 144 |
| 17 | FDCR 1 protein:ab,ti | 1 |
| 18 | #12 OR #13 OR #14 OR #15 OR #16 OR #17 | 14,845 |
| 19 | #11 AND #18 | 106 |
