# Supplementary material for: Prognostic effect of osteoprotegerin in patients with ischemic stroke: A systematic review and meta-analysis
Source: PLoS One. 2024 May 31;19(5):e0303832. doi: 10.1371/journal.pone.0303832 (PMC11142426; doi:10.1371/journal.pone.0303832)
Supplement: S2 Table — (DOCX) [file pone.0303832.s003.docx]

S2 Table. Searching procedure on PubMed

| Step | Query | Results |
| --- | --- | --- |
| 1 | ischemic stroke [MeSH] | 10,853 |
| 2 | ischemic stroke [tiab] | 65,020 |
| 3 | ischemic strokes [tiab] | 3,931 |
| 4 | ischaemic stroke [tiab] | 8,908 |
| 5 | ischaemic strokes [tiab] | 674 |
| 6 | cerebral infarction [MeSH] | 36,412 |
| 7 | cerebral infarction [tiab] | 18,151 |
| 8 | cerebral infarctions [tiab] | 1,601 |
| 9 | brain infarction [MeSH] | 42,769 |
| 10 | brain infarction [tiab] | 2,787 |
| 11 | brain infarctions [tiab] | 375 |
| 12 | #1 OR #2 OR #3 OR #4 OR #5 OR #6 OR #7 OR #8 OR #9 OR #10 OR #11 | 120,493 |
| 13 | osteoprotegerin [MeSH] | 5,189 |
| 14 | osteoprotegerin [tiab] | 6,782 |
| 15 | osteoclastogenesis inhibitory factor [tiab] | 116 |
| 16 | tumour necrosis factor receptor 11b [tiab] | 71 |
| 17 | follicular dendritic cell derived receptor 1 [tiab] | 1 |
| 18 | FDCR 1 protein [tiab] | 1 |
| 19 | #13 OR #14 OR #15 OR #16 OR #17 OR #18 | 8,082 |
| 20 | #12 AND #19 | 35 |
