# Supplementary material for: Prognostic effect of osteoprotegerin in patients with ischemic stroke: A systematic review and meta-analysis
Source: PLoS One. 2024 May 31;19(5):e0303832. doi: 10.1371/journal.pone.0303832 (PMC11142426; doi:10.1371/journal.pone.0303832)
Supplement: S3 Table — (DOCX) [file pone.0303832.s004.docx]

S3 Table. Searching procedure on the Cochrane Library

| Step | Query | Results |
| --- | --- | --- |
| 1 | ischemic stroke: [MeSH] explode all trees | 948 |
| 2 | ischemic stroke: ti,ab,kw | 18,809 |
| 3 | ischemic strokes: ti,ab,kw | 18,809 |
| 4 | ischaemic stroke: ti,ab,kw | 18,809 |
| 5 | ischaemic strokes: ti,ab,kw | 18,809 |
| 6 | cerebral infarction: [MeSH] explode all trees | 1,491 |
| 7 | cerebral infarction: ti,ab,kw | 6,552 |
| 8 | cerebral infarctions: ti,ab,kw | 6,551 |
| 9 | brain infarction: [MeSH] explode all trees | 1,932 |
| 10 | brain infarction: ti,ab,kw | 5,569 |
| 11 | brain infarctions: ti,ab,kw | 5,569 |
| 12 | #1 OR #2 OR #3 OR #4 OR #5 OR #6 OR #7 OR #8 OR #9 OR #10 OR #11 | 25,008 |
| 13 | osteoprotegerin: [MeSH] explode all trees | 155 |
| 14 | osteoprotegerin: ti,ab,kw | 433 |
| 15 | osteoclastogenesis inhibitory factor: ti,ab,kw | 3 |
| 16 | tumour necrosis factor receptor 11b: ti,ab,kw | 3 |
| 17 | follicular dendritic cell derived receptor 1: ti,ab,kw | 1 |
| 18 | FDCR 1 protein: ti,ab,kw | 0 |
| 19 | #13 OR #14 OR #15 OR #16 OR #17 OR #18 | 440 |
| 20 | #12 AND #19 | 7 |
