# Supplementary material for: Prognostic effect of osteoprotegerin in patients with ischemic stroke: A systematic review and meta-analysis
Source: PLoS One. 2024 May 31;19(5):e0303832. doi: 10.1371/journal.pone.0303832 (PMC11142426; doi:10.1371/journal.pone.0303832)
Supplement: S4 Table — (DOCX) [file pone.0303832.s005.docx]

# S4 Table. Quality Assessment of Included Studies by Newcastle-Ottawa Scales

| Study |  | Selection | | | | Comparability | Outcome | | | Total Score |
| --- | --- | --- | --- | --- | --- | --- | --- | --- | --- | --- |
|  |  | Exposed Cohort | Nonexposed Cohort | Ascertainment of Exposure | Outcome of Interest |  | Assessment of Outcome | Length of Follow-up | Adequacy of Follow-up |  |
| Song et al 2012 |  | * | * | * | * | * | * | * | * | 8 |
| Zhu et al 2023 |  | — | * | * | * | * | * | * | * | 7 |
| Wajda et al 2019 |  | — | * | * | * | * | * | * | * | 7 |
| Jensen et al 2009 |  | — | * | * | * | * | * | * | * | 7 |
| Park et al 2022 |  | — | * | * | * | * | * | * | * | 7 |
| Single asterisk indicates 1 score,double asterisk indicates 2 scores,and dash indicates 0 scores. | | | | | | | | | |  |
